# A survey on laypersons' willingness in performing cardiopulmonary resuscitation

**DOI:** 10.1186/cc9715

**Published:** 2011-03-11

**Authors:** T Otani, S Ohshimo, T Shokawa, K Nishioka, J Itai, T Sadamori, Y Kida, T Inagawa, Y Torikoshi, K Suzuki, K Ota, T Tamura, R Tsumura, Y Iwasaki, N Hirohashi, K Tanigawa

**Affiliations:** 1 Hiroshima University, Hiroshima, Japan

## Introduction

Although bystander cardiopulmonary resuscitation (CPR) can improve survival from cardiac arrest, the reported prevalence of bystander CPR remains low in most countries. This study was performed to investigate factors affecting laypersons' willingness in performing CPR.

## Methods

Questionnaires including 10 questions regarding personnel backgrounds, knowledge regarding the use of AED, CPR training, willingness in performing CPR, and EMS dispatcher's advice were distributed to citizens who gathered at a ball park stadium, a typical public place in Hiroshima, Japan.

## Results

Ten thousand questionnaires were distributed and a total of 5,956 were collected for analysis. Age distributions of the respondents were: <20 years old: 13%, 20 to 49 years old: 67%, 50 to 69 years old: 16%, >70 years old: 3%. Fifty-seven percent were male. Ninety-one percent had heard of AED; however, only 45% knew how to use it. Forty-nine percent took CPR training before. As for the willingness to perform CPR, 38% answered they would start CPR, 34% would do it if any advice was available. On the other hand, 23% said they were not capable of performing CPR, and 4% were not willing to do it. Of those who were not capable of performing CPR, the reasons included lack of knowledge and/or skills to perform CPR (50%), no previous CPR training (27%), concern over harm to the victims (25%), and lack of confidence to determine cardiac arrest (19%). Of those who were willing to perform CPR, 61% answered they would prioritize rescue breathing over chest compression. In comparison of those with and without previous CPR training or knowledge of the use of AED, significant differences were found in the willingness in performing CPR (88% vs. 58%, *P *< 0.0001; 91% vs. 58%, *P *< 0.0001, respectively) and doing rescue breathing (55% vs. 29%, *P *< 0.0001; 64% vs. 57%, *P *< 0.0001, respectively). Fifty-two percent of the respondents did not know the service of dispatcher-assisted CPR.

## Conclusions

Our study indicated that proper knowledge of CPR, prior CPR training, and onsite bystander CPR assistance may enhance laypersons' willingness in performing CPR. More emphasis should be exerted on the roles of chest compression and the EMS dispatcher assistance in CPR education.

